# Comparison of safety and efficacy of different endovascular treatments for symptomatic intracranial atherosclerotic stenosis: results from a single center

**DOI:** 10.3389/fneur.2025.1539127

**Published:** 2025-03-05

**Authors:** Qiao Lin, Kaiyi Zhong, Xiyue Pan, Congfang Li, Xiaozhen Lu, Naidong Wang

**Affiliations:** ^1^Department of General Medicine, The Affiliated Hospital of Qingdao University, Qingdao, Shandong, China; ^2^Department of Neurology, The Affiliated Hospital of Qingdao University, Qingdao, Shandong, China

**Keywords:** drug-eluding stent, drug-coated balloon, endovascular treatment, in-stent restenosis, bare metal stent

## Abstract

**Background:**

Symptomatic intracranial atherosclerotic stenosis (sICAS) is one of the common causes of ischemic stroke. However, the treatment of sICAS has remained a challenge in the past with unfavorable findings. This study aimed to evaluate the effectiveness and safety of different endovascular treatment methods for sICAS.

**Methods:**

The study involved 154 patients with sICAS who received endovascular treatment at Qingdao University Hospital between January 2021 and October 2023. Based on the characteristics of the lesions, three different types of treatments were performed: bare metal stent group (BMS group), drug-coated balloon group (DCB group), and drug-eluting stent group (DES group). The primary endpoints included the incidence of in-stent restenosis (ISR) in the 6-month, periprocedural complications, the rate of stroke recurrence in the area of the stented artery during the follow-up period, and modified Rankin score (mRS) at discharge, at 1-month, at 3-month, at 6-month of patients after stenting.

**Results:**

The incidence of perioperative complications did not differ significantly between groups (11.3% in the BMS group, 8.0% in the DCB group, and 6.1% in the DES group, *p* = 0.776). All patients (154/154) had successful reperfusion after endovascular treatment. The incidence of stroke during follow-up was 4.5% (7/154), with 5 (7.0%) patients in the BMS group, 1 (2.0%) patient in the DCB group, and 1 (3.0%) patient in the DES group. The restenosis rate in the BMS group [35.2% (25/71)] tended to be higher than that in the DCB group [6.0% (3/50)] and DES group [9.1% (3/33)]. In multivariate logistic regression analysis, endovascular treatment strategy and vessel distribution were significant independent risk factors for ISR within 6 months (*p* < 0.05).

**Conclusion:**

Adverse events and success rates following stent implantation are comparable across therapy groups in individuals with sICAS. When compared to BMS, DES, and DCB reduce the risk of ISR, with the advantages of the DCB appearing to be greater for some high-risk patients with ICAS.

## 1 Introduction

With 2.5 million stroke victims annually, stroke is the leading cause of death worldwide. The burden of stroke is most likely the largest in China. Intracranial atherosclerotic stenosis (ICAS), one of the main causes of ischemic stroke, is closely associated with a high incidence and death rate from stroke (1). Extracranial large artery atherosclerosis may be a common lesion in white persons in Europe and America. In contrast, atherosclerotic stenosis of the major intracranial arteries is found commonly among stroke patients of Asian, black, and Hispanic ancestry (2–4). Consequently, lowering the high incidence of cerebrovascular accidents requires both secondary prevention and efficient treatment (5). Even with aspirin treatment and standard management of vascular risk factors, patients who have recently experienced a transient ischemic attack (TIA) or stroke and have severe stenosis (70–99% of the diameter of a major intracranial artery) are particularly high risk for recurrent stroke in the territory of the stenotic artery (approximately 23% at 1 year) (6, 7). This suggests that medication-only treatment for symptomatic patients with cerebral artery stenosis might not be a practical means of preventing ischemic stroke. When traditional medical treatment is not working for Asian individuals with a high frequency of ICAS, endovascular therapy can be a helpful alternative. For the endovascular treatment of symptomatic intracranial atherosclerotic stenosis (sICAS), the Stenting vs. Aggressive Medical Management for Preventing Recurrent Stroke in Intracranial Stenosis (SAMMPRIS) and the Vitesse Intracranial Stent Study for Ischemic Stroke Therapy (VISSIT) trials did not demonstrate the benefits of endovascular treatment over medical therapy for the endovascular treatment of sICAS, with a high incidence of peri-operative ischemic or hemorrhagic stroke events (8, 9). Improved patient and device selection techniques were used in follow-up studies to lower the peri-operative event rate (10). Through the introduction of devices and improved neuro-interventional techniques, periprocedural complications have been adequately controlled. Intracranial stenting may be safe for use in some patients with sICAS, according to data from the Wingspan Stent System Post Market Surveillance (WEAVE) trial and the Registry Study of Stenting for Symptomatic Intracranial Artery Stenosis in China, which showed periprocedural complication rates of 2.6 and 4.3%, respectively (11–13). However, it has been observed that in-stent restenosis (ISR) caused by neointimal hyperplasia following stent implantation can reach 33%. This is a serious issue that needs to be resolved right once (14, 15). Consequently, it is imperative to prevent ISR in ICAS lesions. Drug-eluting stents (DES) and drug-coated balloons (DCB) can effectively lower the incidence of ISR by releasing antiproliferative drugs at the stenosis site to inhibit endothelial cell proliferation and vascular overactivity, and the initial application of DES and DCB in sICAS has demonstrated better safety and efficacy (16–20). However, the safety and efficaciousness of DES and DCB applied to intracranial arteries remain unclear, and the ideal course of treatment for sICAS patients is still unknown. The purpose of this paper is to evaluate the safety and effectiveness of several endovascular therapy modalities for sICAS to gather additional knowledge for clinical use.

## 2 Materials and methods

### 2.1 Patients and study design

Approved by the University Hospital of Qingdao’s institutional ethics council, this retrospective investigation was conducted at a tertiary stroke center. A total of 154 patients with for sICAS with endovascular recanalization between January 2021 and October 2023 were enrolled. Our inclusion criteria were: (1) patients over 18 years old; (2) patients with ≥70% stenosis of the main trunk of the middle cerebral artery (MCA), intracranial segment of the internal carotid artery (ICA), intracranial segment of the vertebral artery (VA), or basilar artery (BA) was confirmed by digital subtraction angiography (DSA) following Warfarin-Aspirin Symptomatic Intracranial Disease Study (WASID) criteria 21; (3) TIA or stroke in the territory of the target lesion area; (4) continuing, aggravated, or recurrent ischemic neurological deficits despite maximal medical therapy; (5) at least one atherosclerotic risk factor (hypertension, diabetes mellitus, hyperlipidemia, and smoking); (6) a modified Rankin score (mRS) ≤ 3; and (7) the previous TIA or ischemic stroke occurring more than 3 weeks before the endovascular procedures. The exclusion criteria were as follows: (1) non-atherosclerotic stenosis or concurrent intracranial pathology such as vasculitis and arterial dissection; (2) concurrent intracranial tumor, aneurysm, and cerebral arteriovenous malformation; (3) tandem ≥50% stenosis of extracranial carotid or vertebral artery; (4) a concomitant any bleeding disorder; (5) allergies to heparin, aspirin, clopidogrel, metal implants, or narcotic drugs; and (6) intolerance to general anesthesia, comorbidities of malignant tumor or severe liver and kidney dysfunction.

### 2.2 Endovascular procedures

Pre-procedurally, aggressive medical therapy and intensive risk factor management were implemented. This included stringent blood glucose control, cigarette control, aspirin (100 mg/day), clopidogrel (75 mg/day), and atorvastatin calcium (20 mg/day). Endovascular treatments were performed by experienced neurointerventionists. DSA was performed for all patients, and the strategy of endovascular treatment was decided according to the site and characteristics of the target lesions and based on the operators’ experience and preference, and all the procedures were performed under general anesthesia. Technical success was defined as residual stenosis of the target vessel ≤30% after angioplasty.

### 2.3 BMS group and DES group

The selected DES included NOVA (Tianjin Sanoshenchang Company, China) and NANO (Beijing Lepu Company, China), and the BMS included Apollo (Shanghai Microtronics Company, China) and Neuroform EZ (Boston Scientific Corporation, USA). The femoral artery was punctured and cannulated using the Seldinger technique, with a 6F Cook-long arterial sheath. An intermediate catheter was delivered proximal to the target vessel to measure the maximum stenosis rate of the diseased vessel. The stenosis degree was measured according to the criteria of the WASID study, and to observe the collateral circulation compensation. The micro guidewire was carefully passed through the stenotic vessel and placed in the distal vessel to stabilize the guidewire. A balloon of appropriate size was delivered along the micro guidewire to pre-dilate the stenotic vessel, and after dilatation, the balloon was carefully withdrawn under fluoroscopy, and the stenotic vessel was dilated by DSA imaging. The DES or BMS was delivered along the microguidewire, and the balloon was slowly pressurized with a pressure of 6–8 atm after accurate alignment. After the successful release of the stent, angiography was performed again to observe the residual stenosis of the target vessel and the blood flow status of the vessel after stent implantation. The diameter of the stent was chosen to be slightly smaller than the diameter of the adjacent normal vessel, and the length of the stent was chosen to cover at least 1–2 mm of both ends of the stenotic vessel.

### 2.4 DCB group

A 6F shuttle sheath was inserted under systemic heparinization into the intended artery. Following measurements of the stenotic segment length and the artery’s diameter proximal or distal to the lesion, the dilatation and DCB catheter sizes were established. The balloon’s diameter has to be between 10 and 20% lower than the non-diseased artery’s diameter either proximal or distal to the stenotic segment to prevent arterial rupture. After the intermediate catheter was positioned, a 0.014-inch guidewire was steered through the stenotic segment into the distal circulation. After the wire was traced to the stenosis region, a quick exchange coronary balloon catheter was inflated until the nominal pressure was attained in 30 s. After another 30 s, the balloon catheter was deflated. Following angioplasty with a DCB was done right away if there was no severe dissection or considerable residual stenosis because of either calcified lesions or elastic recoil. The DCB catheter (SeQuent Please, B Braun, Melsungen, Germany) was navigated via the wire to cover the whole diseased segment. After gradually increasing to the nominal pressure, the DCB was left inflated for 60 s. Angiography was done both immediately following the DCB’s deflation and 15–20 min later to make sure there had been no increasing dissection, thrombus formation, or arterial rebound. If there is still a relatively serious residual stenosis rate or arterial dissection and blood flow instability after drug balloon dilation, stent angioplasty can be selected according to the intraoperative situation. Residual stenosis, perforator vessel occlusion, and distal perfusion were evaluated by postoperative angiography.

### 2.5 Periprocedural management

Immediately following surgery, a CT scan was done to rule out brain bleeding. Furthermore, all patients were evaluated with Transcranial Doppler (TCD) for immediate restenosis and hyper-perfusion syndrome (HPS) within 24 h following the surgery. Antihypertensive medications were used to keep blood pressure between 110 and 130/70 and 80 mmHg to avoid HPS. If the patient experienced worsening symptoms after surgery, or if any new ones surfaced, a head Magnetic Resonance Imaging (MRI) would be performed to determine whether the distant embolism was the cause. Oral administration of aspirin 100 mg/day and clopidogrel 75 mg/day was taken for 3 months following the procedure; clopidogrel was discontinued at the 3-month mark, and aspirin 100 mg/day was taken orally for long-term use. Rehabilitation treatment was recommended for patients with functional disability. Long-term management of individual medical risk factors such as blood pressure, cholesterol, and diabetes mellitus were implemented.

### 2.6 Patient follow-up and outcome measures

All patients were followed up regularly through hospitalization or outpatient visits. Six months after the procedure, DSA was taken out to assess ISR, Stenosis >50% of the luminal diameter was considered angiographic restenosis on DSA. With a total score of 0–6, larger scores denoting worse neurological performance, the mRS was used to evaluate functional outcomes at discharge and at the 1-, 3-, and 6-month follow-up (21). The following was the result endpoint: (1) the 6-month incidence of ISR; (2) peri-procedural complications 7 days post-revascularization, including branch embolization, ischemic stroke, stent thrombosis, high perfusion, or symptomatic hemorrhage; (3) the rate of stroke recurrence in the area of the stented artery during the follow-up period. Recurrent ischemic stroke was considered to be any focal neurological symptom of sudden onset that lasted for at least 24 h, was related to the corresponding vascular territory, was not associated with a hemorrhage on brain CT or MRI, and occurred within the follow-up period; and (4) mRS at discharge, at 1-month, at 3-month, at 6-month of patients after stenting. The incidence of ISR in the 6-month was taken as the main prognostic indicator.

### 2.7 Statistical analysis

Continuous variables were described by median (standard deviation) and interquartile range (interquartile range). The differences between groups were determined by a one-way analysis of variance or Kruskal-Wallis test. The count (n) and percentage (%) were used to describe categorical variables, for which the chi-square test or Fisher’s exact tests were used. The impact of the various clinical factors on each outcome node was examined using univariate logistic analysis. If the probability value for the bivariate relationship with the endpoint was less than 0.1, the factor was considered for multivariate logistic regression analysis. Compute the 95% confidence intervals (CI) after that. All analyses were performed using SPSS statistical software version 29.0 (SPSS Inc., Chicago, IL, USA) and R statistical software version 4.0.3 (R Foundation), Graphpadrism9.4 software for drawing. A two-sided *p*-value < 0.05 was considered statistically significant.

## 3 Results

### 3.1 Baseline characteristics

Between January 2021 and October 2023, endovascular treatment (EVT) was used to treat 154 patients (91 males, aged 60.7 ± 8.70) who had TIA or stroke due to severe atherosclerotic stenosis of the intracranial artery. The stenoses were located as follows: intracranial internal carotid artery (ICA; *n* = 25, 16.2%), middle cerebral artery (*n* = 76, 49.4%), basilar artery (*n* = 27, 17.5%), intracranial vertebral artery (*n* = 26, 16.9%) (Table 1). Based on the location and characteristics of the target lesions as well as the experience and choice of the operators, different endovascular therapy techniques were selected. 71 (46.1%) of the 154 patients received BMS treatment, 50 (32.5%) received DCB treatment, and 33 (21.4%) received DES treatment. Intracranial artery stenosis and poor collaterals were all confirmed by DSA before stenting. The clinical risk factors for stroke, characteristics of the target artery, and mRS scores of all enrolled patients were evenly distributed among the three treatment groups, as shown in Table 2. The most common risk factor was hypertension 97 (63.0%), followed by diabetes mellitus 66 (42.9%). 13.6% of patients have a family history of stroke and 31.2% of patients have previously experienced stroke or TIA. There was no significant difference in functional scale, common risk factors, or relative disease history between the three groups.

**Table 1:** Vessels distribution.

	Intracranial VA (*n* = 26)	BA (*n* = 27)	MCA (*n* = 76)	Intracranial ICA (*n* = 25)	Total (*n* = 154)
BMS group, n (%)	15 (21.1)	7 (9.9)	38 (53.5)	11 (15.5)	71 (46.1)
DCB group, n (%)	6 (12.0)	13 (26.0)	21 (42.0)	10 (20.0)	50 (32.5)
DES group, n (%)	5 (15.2)	7 (21.2)	17 (51.5)	4 (12.1)	33 (21.4)

VA, Vertebral artery; BA, Basilar artery; MCA, Middle cerebral artery; ICA, Internal carotid artery; BMS, Bare metal stent; DCB, Drug-coated Balloon; DES, Drug-eluting stent.

**Table 2:** Baseline characteristics of patients receiving endovascular treatment in different groups.

	BMS group (*n* = 71)	DCB group (*n* = 50)	DES group (*n* = 33)	Total	*p*
Sex: male (%)	43 (60.6)	27 (54.0)	21 (63.6)	91 (59.1)	0.643
Age in years [median (IQR)]	59.5 (8.99)	60.7 (9.03)	60.6 (8.14)	60.7 (8.70)	0.433
BMI [median (IQR), kg/m^2^]	26.0 (23.5, 27.7)	25.4 (23.6, 28.0)	25.6 (22.6, 27.4)	25.7 (23.5, 27.7)	0.563
Length of stay [median (IQR), d]	11 (8, 14)	10 (8, 14)	9 (7, 12)	10 (8, 14)	0.098
Alcohol drinking (%)	18 (25.4)	10 (20.0)	10 (30.3)	38 (24.7)	0.558
Smoking history (%)	28 (39.4)	14 (28.0)	12 (36.4)	54 (35.1)	0.424
History of stroke (%)	24 (33.8)	13 (26.0)	11 (33.3)	48 (31.2)	0.630
Family history of stroke (%)	11 (15.5)	6 (12.0)	4 (12.1)	21 (13.6)	0.867
Diabetes mellitus (%)	33 (46.5)	22 (44.0)	11 (33.3)	66 (42.9)	0.443
Hypertension (%)	46 (64.8)	32 (64.0)	19 (57.6)	97 (63.0)	0.765
Atrial fibrillation (%)	1 (1.4)	1 (2.0)	0 (0.0)	2 (1.2)	0.789
Coronary disease (%)	9 (12.7)	9 (18.0)	3 (9.1)	21 (13.6)	0.484
Myocardial infarction (%)	3 (4.2)	1 (2.0)	0 (0.0)	4 (2.6)	0.556
Low-density lipoprotein-cholesterol [mean (SD), mg/dL]	1.98 (0.78)	2.07 (0.73)	1.96 (0.52)	2.00 (0.71)	0.714
High-density lipoprotein-cholesterol [mean (SD), mg/dL]	1.02 (0.21)	1.05 (0.29)	1.05 (0.29)	1.04 (0.25)	0.770
Triglyceride [mean (SD), mg/dL]	1.34 (0.61)	1.24 (0.59)	1.37 (0.90)	1.31 (0.67)	0.627
Total cholesterol [mean (SD), mg/dL]	3.53 (0.96)	3.60 (0.93)	3.53 (0.72)	3.44 (0.90)	0.896
Serum uric acid [mean (SD), mg/dL]	334.2 (90.35)	321.2 (91.17)	314.9 (82.40)	331.6 (88.76)	0.554
Total surgical time [median (IQR), min]	90 (69, 120)	90 (70, 100)	90 (70, 105)	90 (70, 108.5)	0.433
Stenosis length [median (IQR), mm]	6 (6, 8)	6 (6, 8)	6 (6, 8)	6 (6, 8)	0.576
Stenosis [median (IQR), %]	90 (80, 90)	86 (80, 90)	88 (80, 90)	90 (80, 90)	0.372
Residual stenosis [median (IQR), %]	15 (15, 20)	12.5 (10, 20)	10 (10, 20)	15 (10, 20)	0.224
mRS at discharge [median (IQR)]	1 (0, 1)	1 (0, 1)	1 (0.5, 1)	0 (0, 1)	0.617

BMI, body mass index; TIA, transient ischemic attack; mRS, Modified Rankin Scale; Data presentation, continuous data were presented as mean (standard deviation) if in the normal distribution, but as median [interquartile range (IQR)] if not in the normal distribution, other classification data are expressed as n (%); BMS, Bare metal stent; DCB, Drug-coated Balloon; DES, Drug-eluting stent.

### 3.2 Perioperative outcome

The perioperative outcome is displayed in Table 3. 100% of patients (154/154) had effective reperfusion following EVT. Eight patients [16.0%(8/50)] in the DCB group experienced dissection following dilatation, but forward blood flow remained unaffected. The surgery was terminated after 10 min of monitoring when follow-up angiography revealed that the forward blood flow was still unaffected. None of the eight patients who experienced dissection showed any related ischemic symptoms. There were no patients who underwent salvage stent placement due to flow restriction dissection after DCB expansion. Specifically, six patients in the BMS group developed symptomatic intracerebral hemorrhage, one patient experienced an ischemic stroke in the branch area, and one patient underwent branch embolization. Within the DCB group, one patient developed stent thrombosis within 24 h of EVT, two patients suffered symptomatic intracerebral hemorrhage, and one patient underwent symptomatic hemorrhagic transformation due to hyper-reperfusion. Two patients in the DES group had symptomatic intracerebral bleeding and one patient had branch embolization. The incidence of peri-procedural complications was not shown to have significant differences across the groups (*p* = 0.776; 11.3% in the BMS group, 8.0% in the DCB group, and 6.1% in the DES group).

**Table 3:** Perioperative outcome of patients who received EVT.

	BMS group (*n* = 71)	DCB group (*n* = 50)	DES group (*n* = 33)	Total (*n* = 154)	*p*
Postprocedural perfusion					
TICI = 2b (n, %)	4 (5.6)	4 (8.0)	0 (0.0)	8 (5.2)	0.225
TICI = 3 (n, %)	67 (94.4)	46 (92.0)	33 (100.0)	146 (94.8)	0.225
Complication rate	8 (11.3)	4 (8.0)	2 (6.1)	14 (9.1)	0.776
Branch embolization (n, %)	1 (1.4)	0 (0.0)	1 (3.0)	2 (1.3)	0.488
Symptomatic hemorrhage (n, %)	6 (8.5)	2 (4.0)	1 (3.0)	9 (5.8)	0.491
Ischemic stroke (n, %)	1 (1.4)	0 (0.0)	0 (0.0)	1 (0.6)	1.000
Stent thrombosis (n, %)	0 (0.0)	1 (2.0)	0 (0.0)	1 (0.6)	0.539
High perfusion (n, %)	0 (0.0)	1 (2.0)	0 (0.0)	1 (0.6)	0.539

EVT, endovascular treatment; BMS, Bare metal stent; DCB, Drug-coated Balloon; DES, Drug-eluting stent; mTICI, modified thrombolysis in cerebral ischemia scale.

### 3.3 Follow-up outcome

All patients received clinical and imaging follow-ups for more than 6 months. Table 4 showed that stroke occurred at a rate of 4.5% (7/154) during follow-up, five patients (7.0%) in the BMS group, one patient (2.0%) in the DCB group, and one patient (3.0%) in the DES group. The 30-day mRS scores, 90-day mRS scores, and 180-day mRS scores showed no significant difference among the three groups. During follow-up, all patients received DSA. The rate of restenosis in the BMS group [35.2%(25/71)] tended to be higher than that in the DCB group [6.0% (3/50)] and the DES group [9.1%(3/33)], and there was a significant difference among the three groups. In the DES group, there was an approximately 50% higher rate of restenosis (3 [9.1%] vs. 3 [6.0%]) compared with the DCB group, with no significant difference.

**Table 4:** 6-month follow-up complications.

	BMS group (*n* = 71)	DCB group (*n* = 50)	DES group (*n* = 33)	Total (*n* = 154)	*p*
30-day mRS score [median (IQR)]	0 (0, 1)	0 (0, 1)	0 (0, 1)	0 (0, 1)	0.617
90-day mRS score [median (IQR)]	0 (0, 1)	0 (0, 1)	0 (0, 1)	0 (0, 1)	0.445
180-day mRS score [median (IQR)]	0 (0, 1)	0 (0, 1)	0 (0, 1)	0 (0, 1)	0.411
Stroke recurrence within 6 months (%)	5 (7.0)	1 (2.0)	1 (3.0)	7 (4.5)	0.536
Restenosis (%)	25 (35.2) *	3 (6.0)	3 (9.1) **	31 (20.1)	<0.001

mRS, modified Rankin Scale; BMS, Bare metal stent; DCB, Drug-coated Balloon; DES, Drug-eluting stent; *p-value < 0.001 compared with DCB group; **p-value = 0.005 compared with BMS group.

### 3.4 Logistic regression analysis

**Figure 1:**
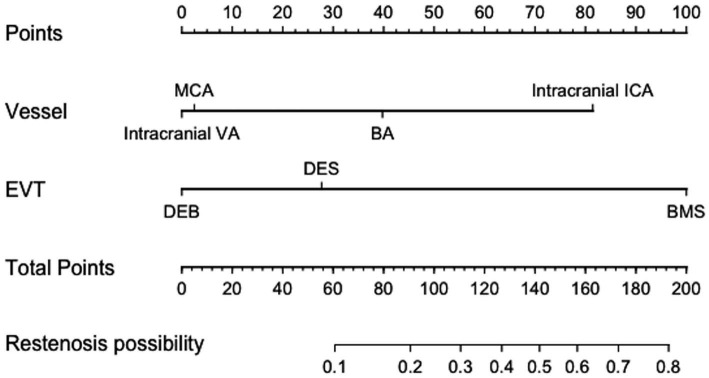
Nomogram predicting restenosis probability within 6 months after EVT. EVT, endovascular treatment; VA, Vertebral artery; BA, Basilar artery; MCA, Middle cerebral artery; ICA, Internal carotid artery; EVT, endovascular treatment; BMS, Bare metal stent; DCB, Drug-coated Balloon; DES, Drug-eluting stent.

Table 5 displays the findings of the univariate analysis of the perioperative and follow-up outcomes. The results of univariate analyses indicated that symptomatic hemorrhage during the perioperative period was related to length of stay, smoking history, alcohol history, total surgical time, and stenosis length; on the other hand, risk factors for the perioperative complications rate included length of stay, smoking history, triglyceride, total surgical time, and stenosis length. While the distribution of vessels and the method of endovascular therapies were linked to restenosis, the recurrence of stroke was related to coronary disease, low-density lipoprotein cholesterol, triglycerides, total cholesterol, and serum uric acid. Multivariate logistic regression analysis revealed that the endovascular treatment strategy, the distribution of vessels for restenosis, the length of stay and total surgical time for perioperative complications, and total surgical time for symptomatic hemorrhage were significant (*p* < 0.05) independent risk factors. The only independent risk factor for stroke recurrence within 6 months was coronary disease (Table 6). Also, it was discovered that these two factors were significant when used in the multivariable model of restenosis probability. A nomogram predicting restenosis probability within 6 months after EVT was constructed with these two parameters based on the multivariable model (Figure 1).

**Table 5:** Univariate logistic analyses for risk factors.

	OR (95%CI)			
	Symptomatic hemorrhage	Perioperative Complication rate	Restenosis within 6 months	Stroke recurrence within 6 months
Sex	0.708 (0.170–2.944)	1.092 (0.360–3.317)	1.054 (0.474–2.344)	1.087 (0.235–5.036)
Age	0.980 (0.909–1.057)	1.004 (0.943–1.069)	1.020 (0.974–1.068)	1.049 (0.956–1.150)
Length of stay	1.214 (1.087–1.355) *	1.173 (1.067–1.290) *	0.979 (0.900–1.065)	0.827 (0.640–1.070)
Alcohol drinking	4.242 (1.077–16.710) *	2.531 (0.818–7.833)	1.078 (0.437–2.662)	1.233 (0.229–6.633)
Smoking history	7.298 (1.460–36.491) *	2.725 (0.893–8.315) *	1.023 (0.449–2.332)	1.412 (0.304–6.552)
History of stroke	1.836 (0.471–7.166)	2.415 (0.796–7.320)	1.065 (0.458–2.479)	1.700 (0.365–7.910)
Family history of stroke	1.895 (0.366–9.805)	1.061 (0.220–5.118)	1.728 (0.610–4.898)	1.058 (0.121–9.259)
Diabetes mellitus	0.651 (0.157–2.704)	1.000 (0.330–3.035)	1.324 (0.600–2.918)	1.828 (0.395–8.461)
Hypertension	2.139 (0.429–10.667)	2.302 (0.614–8.629)	1.086 (0.478–2.470)	3.692 (0.433–31.477)
BMI	1.029 (0.821–1.289)	0.972 (0.806–1.173)	0.983 (0.860–1.123)	1.052 (0.817–1.354)
Coronary disease		0.462 (0.057–3.725)	1.286 (0.432–3.832)	10.196 (2.100–49.501) *
Low-density lipoprotein-cholesterol	0.673 (0.228–1.991)	0.776 (0.334–1.800)	0.979 (0.561–1.707)	2.156 (0.941–4.939) *
High-density lipoprotein-cholesterol	0.421 (0.023–7.709)	0.236 (0.20–2.814)	0.868 (0.179–4.202)	0.223 (0.007–6.995)
Triglyceride	1.478 (0.643–3.397)	1.781 (0.920–3.450) *	1.379 (0.803–2.367)	2.756 (1.256–6.046) *
Total cholesterol	0.678 (0.290–1.586)	0.761 (0.390–1.483)	1.006 (0.649–1.560)	1.940 (0.962–3.911) *
Serum uric acid	0.999 (0.992–1.007)	0.999 (0.993–1.006)	1.002 (0.997–1.006)	1.007 (0.999–1.016) *
Total surgical time	1.024 (1.008–1.041) *	1.017 (1.004–1.030) *	1.008 (0.997–1.018)	0.985 (0.955–1.016)
Stenosis length	2.465 (1.319–4.610) *	1.857 (1.184–2.913) *	0.952 (0.709–1.279)	0.628 (0.341–1.157)
Residual stenosis	1.052 (0.940–1.177)	0.988 (0.898–1.087)	0.970 (0.905–1.039)	1.000 (0.887–1.139)
Endovascular options				
BMS	–	–	–	–
DCB	0.451 (0.087–2.334)	0.685 (0.194–2.412)	0.117 (0.033–0.416) *	0.269 (0.030–2.380)
DES	0.339 (0.039–2.932)	0.508 (0.102–2.537)	0.184 (0.051–0.664) *	0.412 (0.046–3.679)
Distribution of vessels				
VA	–	–	–	–
BA	–	0.960 (0.125–7.371)	1.250 (0.296–5.284)	3.125 (0.304–32.165)
ICA	–	1.636 (0.250–10.728)	4.321 (1.147–16.276) *	–
MCA	–	1.217 (0.236–6.268)	0.931 (0.269–3.224)	1.027 (0.102–10.333)

OR, odds ratio; CI, confidence interval; TIA, transient ischemic attack; BMI, body mass index; VA, Vertebral artery; BA, Basilar artery; MCA, Middle cerebral artery; ICA, Internal carotid artery; BMS, Bare metal stent; DCB, Drug-coated Balloon; DES, Drug-eluting stent. *Indicates *p*-value < 0.1 in Univariate logistic analyses.

**Table 6:** Predictors of outcome (multivariate analysis).

Clinical outcome	Factors	OR (95%CI)	*p*
Symptomatic hemorrhage	Length of stay	1.177 (0.991–1.397)	0.064
	Smoking history	7.145 (0.411–124.204)	0.177
	Alcohol drinking	2.420 (0.170–34.532)	0.515
	Stenosis length	2.235 (0.937–5.329)	0.070
	Total surgical time	1.034 (1.014–1.055)	0.001
Perioperative complication rate	Length of stay	1.156 (1.024–1.304)	0.019
	Smoking history	2.350 (0.633–8.729)	0.202
	Triglyceride	1.326 (0.501–3.510)	0.570
	Total surgical time	1.017 (1.003–1.031)	0.015
	Stenosis length	1.559 (0.955–2.545)	0.076
Restenosis rate	Endovascular options		
	BMS	–	<0.001
	DCB	0.067 (0.016–0.272)	<0.001
	DES	0.141 (0.035–0.565)	0.006
	Distribution of vessels		
	VA	–	0.005
	BA	2.937 (0.588–14.664)	0.189
	ICA	9.082 (1.958–42.132)	0.005
	MCA	1.069 (0.291–3.933)	0.920
Stroke rate within 6 months	Coronary disease	14.736 (2.135–101.727)	0.006
	Low-density lipoprotein-cholesterol	0.168 (0.001–22.364)	0.474
	Triglyceride	2.341 (0.943–5.810)	0.067
	Total cholesterol	4.577 (0.084–250.095)	0.456
	Serum uric acid	1.009 (0.999–1.019)	0.090

OR, odds ratio; CI, confidence interval; VA, Vertebral artery; BA, Basilar artery; MCA, Middle cerebral artery; ICA, Internal carotid artery; BMS, Bare metal stent; DCB, Drug-coated Balloon; DES, Drug-eluting stent.

## 4 Discussion

In this study, we retrospectively investigated the safety and efficacy of different endovascular treatments for sICAS. Our research shows that: (1) the perioperative complication rate of patients with symptomatic intracranial atherosclerotic stenosis is 9.1, 4.5% of patients have at least one ischemic stroke in the ipsilateral intracranial artery area during the 6-month follow-up, and there is no significant difference in perioperative complications, improvement of cognitive function, and recurrent stroke within 6 months among the three groups; (2) In our study, the incidence of ISR in DES and DCB was lower than that in BMS, with statistical difference. The incidence of ISR in DCB was lower than that in BMS, although there was no statistical difference between the two groups. DCB is more likely to be the optimal treatment strategy.

Given the unfavorable outcomes of the SAMMPRIS and VISSIT studies, concerns have been raised regarding the safety and effectiveness of endovascular therapy for intracranial atherosclerotic stenosis. According to the SAMMPRIS study, the stenting arm using the Wingspan stent had a higher rate of 30-day stroke and death (14.7%) compared to the medical arm (12.6%), and a higher rate of 1-year stroke and death (19.7%) compared to the aggressive medical therapy (5.8%) (22). The VISSIT trial showed that the stenting group with a BMS had an even higher 30-day stroke/hard TIA rate (24.1%) compared to the medical group (9.4%) and that the stenting group’s 1-year stroke/hard TIA rate (36.2%) was much higher than the medical group’s (15.1%) (9). However, owing to the limitations of the trials, EVT is still seen by researchers and clinicians as a potentially effective way to prevent stroke in individuals with sICAS. Compared with the SAMMPRIS study, the incidence of perioperative complications in the bare stent group, drug-coated balloon group, and drug-coated stent group in this study were 11.3, 8.0, and 6.1%, respectively, all lower than 14.7%. The main reason could be that the surgeons in this study have a wealth of clinical experience, are more proficient at technical operations, and have a lower frequency of issues such as perforator obstruction and arterial dissection. Other reasons are strict preoperative screening and perioperative care, postoperative monitoring, stringent patient blood pressure and blood sugar control, and postoperative medication. Every patient group’s prognosis remains favorable throughout the follow-up procedure, and no deaths occur. Drug delivery devices such as drug-coated balloons and drug-eluting stents have been introduced in recent years. These devices attach drugs that prevent cell proliferation to their surface and release the drugs evenly onto the vascular wall. This reduces the risk of restenosis, endometrial hyperplasia, and cell proliferation. In this study, the incidence of 6-month restenosis was found to be 35.2% in the BMS group, 6.0% in the DCB group, and 9.1% in the DES group. Notably, the restenosis rate in the BMS group was significantly greater than that of the other two groups. In 2021, the results of the WANG et al. study showed that the incidence of perioperative complications was 2.9%, and the incidence of restenosis was 12%. The results of the RE-MONDA et al. study showed that the incidence of perioperative complications was 0%, and the incidence of restenosis 9 months after surgery was 12%. DCB treatment for sICAS has a decreased rate of restenosis, according to research findings, despite potential differences in the study’s design and use of DCB. It can be seen that DCB has a good effect in improving intracranial arterial ISR problems. Likewise, DES is beneficial in resolving intracranial artery ISR problems. In 2022, the results of the Jia et al. study showed that the incidence of perioperative complications was 7.6%, and the incidence of restenosis was 9.5% (17). Currently, there are few comparable studies of DCB and DES for symptomatic ICAS. In this study, the restenosis rate of the DES group was approximately 50% higher than that of the DCB group (3 [9.1%] vs. 3 [6.0%]), although there was no significant difference between the two groups. Compared to DES, DCB has greater pass ability, can increase the immediate success rate of surgery, and medication concentration at the lesion site, minimizes the danger of branch occlusion, prevents chronic inflammatory reactions of metal trabeculae and polymers due to the absence of stent foreign bodies (23, 24). On the other hand, DCB does not use a permanent implant, which lowers the risk of stent-related unfavorable biological reactions that cause thrombosis and restenosis, and promotes the vessel’s beneficial natural healing process (25–27). In addition to providing an antiproliferative medication, DCB facilitates mechanical expansion, which leads to positive vessel remodeling marked by the enlargement of the late lumen, the reduction of plaque, and the stabilization of plaque (28, 29). At the same time, DCB prevents foreign body placement and preserves the opportunity for subsequent treatment when necessary for the patient.

In addition, we found that under the same conditions, the restenosis rate of the intracranial internal carotid artery was higher than that of other intracranial vessels at a 6-month follow-up. Possible reasons are the intracranial ICA segments are more tortuous than other intracranial arteries, which can complicate endovascular device navigation or cause the stent to not fully expand during deployment, which can cause thrombus formation. The low adhesion ability of stents in tortuous arteries may cause more severe intimal hyperplasia, which in turn could cause more severe internal carotid artery stent restenosis. Because there are fewer tenuous perforators in the intracranial ICA, there are fewer problems from perforator obstruction or damage. Postoperative high perfusion was observed in only one patient in our study, and that patient’s case involved the intracranial internal carotid artery. One possible explanation for this could be the ICA being considerably larger than other intracranial arteries such as the middle cerebral artery, which can easily cause high perfusion syndrome after the stenotic lumen is resolved by stenting.

Vessel toxicity is a theoretical risk for DES and DCB that can be caused by the brain’s unique structural and functional complexity, as well as the specific response of cerebral blood vessels to drugs (30). A study that investigated the safety of drug-eluting DES in dog’s BA artery reveals no neurotoxic effects were observed in the intracranial vessel walls or brainstem tissue in which sirolimus-coated stents were implanted (31). Sun et al. (32) reported a long-term safety assessment of DES shows a safety profile similar to BMS, without any neurotoxic histological signs. Xu et al. (33) observed that rapamycin-eluting balloon is a safe and effective treatment for ICAS, respectively, and did not observe any sign of the toxic effect. We have checked arterial and cerebral toxicity by angiography and clinical status, respectively, and did not observe any sign of toxic effect. In addition, there are no reports of neurotoxic complications in other DES and DCB studies applied to cerebral artery stenosis (19, 34, 35). Further studies are needed to confirm whether vascular toxicity occurs.

This study has a few limitations. First, this was a retrospective, single-center, non-randomized study, and suffers from selection bias. Second, the sample size is relatively small for logistic regression analysis. Therefore, a multicenter, prospective, controlled trial is still needed to confirm our results.

## 5 Conclusion

In patients with symptomatic intracranial atherosclerotic stenosis, different treatment groups have similar success rates and adverse events in stent implantation. Compared to bare metal stents, drug-eluting stents, and drug-coated balloons can reduce the risk of ISR, and drug-coated balloons seem to show greater advantages for some high-risk patients with ICAS. However, this needs to be confirmed by further investigation, preferably in large multicenter randomized controlled clinical trials.

## Data Availability

The raw data supporting the conclusions of this article will be made available by the authors, without undue reservation.
